# Apoptosis-inducing effects of jujube (*Zǎo*) seed extracts on human Jurkat leukemia T cells

**DOI:** 10.1186/s13020-016-0085-x

**Published:** 2016-04-01

**Authors:** Natthanan Taechakulwanijya, Natthida Weerapreeyakul, Sahapat Barusrux, Sirithorn Siriamornpun

**Affiliations:** Graduate School, Faculty of Pharmaceutical Sciences, Khon Kaen University, Khon Kaen, 40002 Thailand; Faculty of Pharmaceutical Sciences, Khon Kaen University, Khon Kaen, 40002 Thailand; Faculty of Associate Medical Sciences, Khon Kaen University, Khon Kaen, 40002 Thailand; Department of Food Technology and Nutrition, Mahasarakham University, Mahasarakham, 44000 Thailand

**Keywords:** Jujube, Apoptosis, Jurkat leukemia T cells, Cancer

## Abstract

**Background:**

Jujube (*Zǎo*) seeds exhibited anticancer effects and used in Chinese medicine for many years. This study aims to investigate the apoptosis-inducing effects of seed extracts from eight different cultivated species (‘Apple’, ‘Bombay’, ‘Jumbo’, ‘Kaew’, ‘Nomsod’, ‘Rianthong’, ‘Samros’, and ‘Taiwan’) on human Jurkat leukemia T cells.

**Methods:**

We evaluated the effects of seed extracts from eight jujube cultivated species on human Jurkat leukemia T cells. The crude seed extracts were prepared sequentially by using water, 95 % ethanol, dichloromethane, ethyl acetate, chloroform or hexane. The antiproliferative effects of the jujube seed extracts relative to that of melphalan were evaluated by neutral red assays. Apoptotic cell death induced by the ethanolic extracts at 1 × IC_50_ and 2 × IC_50_ concentrations was demonstrated by DAPI staining, gel electrophoresis, flow cytometry with Annexin V/propidium iodide staining, and caspase-3, -8, and -9 enzyme activities.

**Results:**

Ethanolic extracts of ‘Taiwan’, ‘Jumbo’, ‘Nomsod’, ‘Rianthong’, ‘Samros’, and ‘Bombay’, significantly inhibited the proliferation of Jurkat cells compared with untreated cells (all *P* < 0.001), while the extracts of ‘Kaew’ and ‘Apple’ were inactive. The six active extracts preferentially induced apoptotic cell death in a concentration-dependent manner with DNA fragmentation (2 × IC_50_). Increased caspase-3 activity was detected after treatment with the six extracts. The ‘Taiwan’, ‘Nomsod’, ‘Jumbo’, and ‘Rianthong’ extracts (2 × IC_50_) induced both the extrinsic and intrinsic apoptosis pathways by increasing caspase-8 and caspase-9 activity, respectively. Alkaloids (Dragendorff’s method) and reducing sugars (Fehling’s test) were mainly identified in the apoptosis-inducing extracts.

**Conclusions:**

The tested of six active extracts (‘Taiwan’, ‘Jumbo’, ‘Nomsod’, ‘Rianthong’, ‘Samros’ and ‘Bombay’) contained alkaloids or reducing sugars, and induced caspase-dependent apoptosis in human Jurkat leukemia T cells.

## Background

Complementary and alternative medicines have been evaluated in addition to chemotherapy, radiation, and surgery in treating cancer. The development of an effective strategy for cancer drug discovery has been outlined [1]. Apoptosis is a major target for chemoprevention and chemotherapy [2].

Jujube (*Zǎo*) is a functional food with nutritional value that is consumed in Asia, the Mediterranean and the United States [3–6]. The two extant species include Indian jujube (*Ziziphus mauritiana* Lam.) and Chinese jujube (*Ziziphus jujuba* Mill.) [4], and were reported to exhibit sedative, anodyne, pectoral, stomachic, styptic, and tonic effects [7], as well as anticancer, antianxiolytic, antifungal, and antispastic effects [8].

Different parts of *Z. mauritiana* and *Z. jujuba* exhibit various medicinal effects. *Z. jujuba* has long been used as a crude drug in Chinese medicine [5]. The seeds of *Z. jujuba* enhanced cell membrane permeability for drugs [9] and exhibited sedative effects [10]. Linoleic and stearic acids from seed extracts of *Z. jujuba* significantly inhibited cyclooxygenase (COX)-1 and COX-2 activity [11]. The fruits of *Z. jujuba* activated choline acetyltransferase and increased acetylcholine synthesis [12], through both antioxidant [13, 14] and anti-allergenic [15] effects. Triterpenoids from the leaves of *Z. jujuba* were sweetness inhibitors [16]. The leaves of *Z. mauritiana* protected the liver from alcohol damage [17]. The bark of *Z. mauritiana* possessed both antiulcer [18] and antifertility [19] activities. The roots of *Z. mauritiana* exhibited antifungal [20] and antidiarrheal [21] activities. The functional constituents of jujube possessed anticancer activity [22]. *Z. jujuba* fruits induced apoptosis in liver (HepG2) and breast (MCF-7 and KBR3) cancer cell lines [7, 23]. The seeds of *Z. mauritiana* induced apoptosis in promyelocytic leukemia cells (HL-60) [24].

New jujube hybrids are crossbred from the two varieties (*Z. jujuba* and *Z. mauritiana*). Presently, many new cultivated varieties are available in Thailand and independently exhibit various biological functions [25–27]. Evidence of changes in phytoconstituents between the wild-type plants and new cultivars has been reported [28, 29]. The metabolites found in the leaves of new carrot cultivars possessed higher contents of feruloyl acid, quinic acid, malic acid, and leucine, but lower contents of glucose and sucrose than the wild-type (*Z. mauritiana*) [28]. A new peanut cultivar possessed higher 4α-monomethylsterol contents, but lower 4-desmethylsterol and triterpene alcohol contents than the wild-type plant [29].

This study aims to investigate the apoptosis-inducing effects of seed extracts from eight different jujube cultivated species (‘Apple’, ‘Bombay’, ‘Jumbo’, ‘Kaew’, ‘Nomsod’, ‘Rianthong’, ‘Samros’, and ‘Taiwan’) on human Jurkat leukemia T cells. The anticancer activities were evaluated based on the cytotoxicity and apoptosis-inducing effects on human Jurkat leukemia T cells, relative to melphalan as a positive control. Nuclear morphological changes and caspase-mediated apoptosis were determined. Phytochemical groups in the seed extracts were identified.

## Methods

### Chemicals and reagents

Methanol, isopropanol, dichloromethane, ethyl acetate, chloroform, and hexane were purchased from RCI Labscan Ltd., Thailand, and 95 % ethanol from J.T. Baker^®^ (Center Valley, PA, USA). Sodium hydroxide, citric acid and hydrochloric acid were obtained from BDH Prolabo chemicals (Poole, UK) Neutral red and 4-(p-nitrobenzyl) pyridine (NBP) and acetonitrile were obtained from Sigma-Aldrich^®^ (St. Louis, MO, USA). Melphalan, 4,6-diamidino-2-phenylindole (DAPI), copper (II) sulfate were from Sigma-Aldrich Chemie GmbH (Munich, Germany). Dulbecco’s modified Eagle medium (DMEM), RPMI Media 1640, fetal bovine serum and penicillin streptomycin were from GIBCO, Invitrogen Corp. (Grand Island, NY, USA). Sodium chloride and boric acid were from Vivantis Inc., (Oceanside, CA, USA). Dimethylsullfoxide (DMSO) (molecular grade) was from Sigma-Aldrich Chemie GmbH (Saint-Quentin Fallavier, France). Trypan blue was from Fluka Chemika (Buchs, Switzerland) and potassium sodium tartrate was from Loba Chemie (Mumbai, India). Agarose molecular grade was purchased from Bio-Rad (Hercules, CA, USA). A 100 bp DNA ladder marker was purchased from Invitrogen (Carlsbad, CA, USA). Caspase-Glo^®^ 3/7, 8 and 9 were purchased from Promega (Madison, WI, USA).

### Preparation of crude extracts

**Fig. 1:**
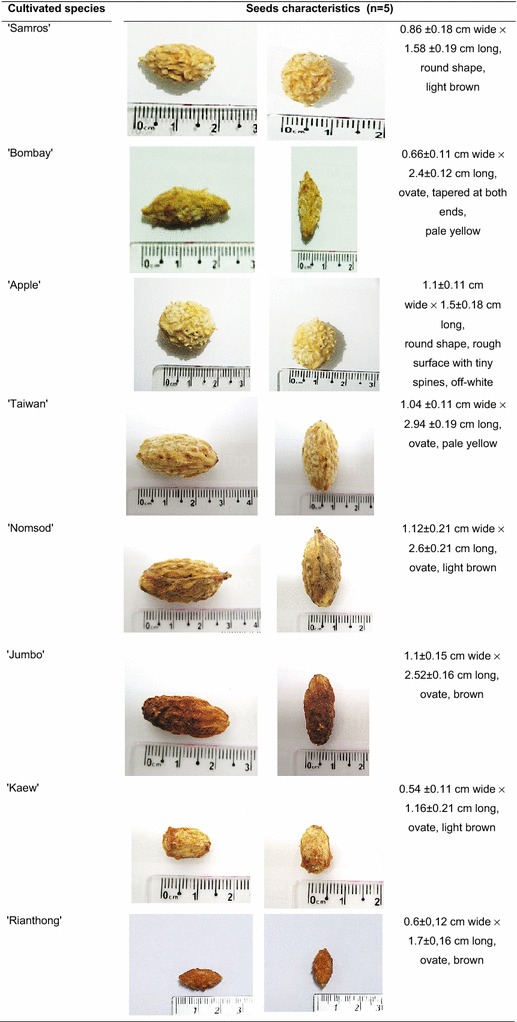
Characteristics of jujube seeds

Eight jujube cultivated species (‘Apple’, ‘Bombay’, ‘Jumbo’, ‘Kaew’, ‘Nomsod’, ‘Rianthong’, ‘Samros’ and ‘Taiwan’) were commercially cultivated and authenticated based on fruit and seed morphological characters by Assistant Professor Thaweesak Thitimetharoch, Division of Pharmacognosy and Toxicology, Faculty of Pharmaceutical Sciences, Khon Kaen University. The jujubes were collected from the local market in Amphoe Muang, Khon Kaen, Thailand, in 2012. The picture vouchers of the specimens (NW-55-2012–NW-62-2012) were deposited at the herbarium of the Faculty of Pharmaceutical Sciences, Khon Kaen University, Khon Kaen province, Thailand. The seed characteristics of each jujube cultivated species (five samples for each) are presented in Fig. 1. The seed was separated and dried at ≤55 °C. The seeds were macerated in deionized water (1 g:3 mL) overnight. The water crude extract was collected after being freeze-dried. The remaining residue from water extraction was dried at 55 °C and further macerated with 95 % ethanol, dichloromethane, ethyl acetate, chloroform or hexane, respectively. Each round of solvent extraction lasted 72 h, using different dry residue to solvent ratios (1 g:3 mL). Solvent was removed by a rotary evaporator (IKA^®^, Staufen, Germany), resulting in a dry residue. Six types of crude extract were obtained for each cultivars. The crude extracts were stored at −20 °C. The concentrations of 10, 25, 50, 100, 250 and 500 µg/mL were used in the antiproliferation study.

### Cell culture

The human Jurkat leukemia T cells were cultured in RPMI 1640 media while the normal African green monkey kidney epithelial cell line (Vero) was cultured in DMEM. Both cell lines were provided by Associate Professor Sahapat Barusrux. Both media were maintained with 10 % fetal bovine serum and 1 % penicillin–streptomycin. Cells were incubated at 37 °C and 5 % CO_2_.

### Antiproliferation assay

The antiproliferation test was determined based on modified neutral red assay [30]. A Jurkat cell density of 5 × 10^5^ cells/mL and Vero 3 × 10^5^ cells/mL were seeded in 96-well plates and maintained at 37 °C for 24 h in humidified 5 % CO_2_. The extracts dissolved in DMSO were pipetted into each well to generate various final concentrations ranging between 10 and 500 µg/mL. The final concentration of DMSO did not exceed 1 % v/v to minimize the cytotoxic effect of DMSO [30]. After treatment with the extracts for 24 h, cells were re-suspended. Neutral red dyes (50 µg/mL) were added to each well. Cells were incubated for another 2 h, then washed with 1 × PBS (pH 7.4) and lysed with 0.33 % HCl in isopropanol. The absorbance of viable cells and dye solution was measured at 537 nm (650 nm reference wavelength) by a microplate reader (TECAN, Grödig, Austria). The untreated cells comprised the control group and cells treated with alkylating drug melphalan are the positive control. A plot of %cell viability and various extract concentrations was used to calculate the IC_50_—which represents the concentration possessing 50 % antiproliferation. The selectivity index (SI) was calculated from the IC_50_ of the extracts in normal Vero cells over the Jurkat cancer cells to indicate the cytotoxic selectivity (i.e., safety) of the crude extracts [31].

### Detection of apoptosis

#### Nuclear morphological alteration

Morphological changes in cell nuclei undergoing apoptosis was determined by DAPI staining [32]. The stained nuclei were then detected by a fluorescent microscope (Nikon Eclipse 80i, Kanagawa, Japan). Cells were seeded in 24-well plates at a density of 3 × 10^5^ cells/mL per well and incubated for 24 h. Cells were treated with 500 µg/mL extracts and incubated for another 24 h. Then cells were washed with PBS and collected by centrifugation (Daihan Scientific, Seoul, Korea) at 1677*g* for 5 min. Cells were fixed in ice-cold methanol for 15 min. After the methanol was removed, the cells were incubated for 30 min, at room temperature, in the dark, with 0.3 µg/mL DAPI. The mixture of PBS to glycerin (at a 1:1 ratio) was added to achieve a 20-µL volume. Cells were wet-mounted on a glass slide and observed under a fluorescent microscope by fluorescence filters with an excitation band of 358 nm and an emission of 461 nm. The images of stained nuclei were captured by the NIS-Element AR 3.2 imaging software (Nikon Instruments Inc, NY, USA).

### Mode of cell death

Flow cytometry was used to determine various types of cell death including early and late stage apoptosis as well as necrosis. Annexin V-FITC and propidium iodide (Annexin V-FITC apoptosis detection kit, eBiosciences, Inc., San Diego, CA, USA) [33]. Cells were seeded in 24-well plates at a density of 5 × 10^5^ cells/ml per well and incubated for 24 h. Cells were treated with the crude extracts at a concentration of 1 × IC_50_ and 2 × IC_50_, obtained from the antiproliferation study at 12 and 24 h. Afterward, cells were harvested by centrifugation (Wisds’ Laboratory Instruments, Korea) at 1677*g* for 5 min then the supernatant was removed. The cells were washed with 200 µL of 1x binding buffer. After removal of the supernatant, 95 µL of binding buffer and 5 µL Annexin V-FITC were added and the mixture incubated in the dark for 15 min at room temperature. Then 95 µL of binding buffer and 5 μL of propidium iodide (final concentration of 2 μg/mL per cell sample) were pipetted into an Eppendrof tube and the mixture incubated for 15 min in the dark at the room temperature. Cells were re-suspended in 200 μL of binding buffer. The stained cells were analyzed immediately by flow cytometry (BD FACSCanto II, Franklin Lakes, NJ, USA) by FACSDiva software version 6.1.3 (BD Biosciences, San Jose, CA, USA).

### Caspases activity

The activity of caspase-3/7, -8 and -9 were evaluated to confirm whether apoptosis was induced by the jujube seed extract(s) and to determine the apoptosis induction pathway. Jurkat cells (1.3 × 10^4^ cells/well) were seeded into 96-well white plates (Costar™, Corning, NY, USA). Cells were incubated at 37 °C for 24 h and extracts added to each well for a final concentration of 2 × IC_50_. Treated cells were incubated at various intervals. Supernatant (50 mL) was pipetted out of each well and 50 µL of caspase reagent mixture added and incubated in the dark for 40 min [34]. The relative luminescence units (RLU) were measured at 562 nm by a Multifunction Microplate Reader (Varioskan™ Flash Multimode Reader, Thermo Scientific, USA) equipped with SkanIt Software 2.4.3 DDE’s program (Thermo Scientific, Waltham, MA USA).

### DNA fragmentation

Late stage apoptotic death mode was confirmed by a DNA fragment assay. Cells (2 × 10^6^ cells/mL density) were seeded in 24-well plates and incubated for 24 h. Cells were treated with the jujube seed extracts as described above in the cell death mode assay. Cells were harvested and washed with PBS. After PBS was removed, 300 µL of lysis buffer (FlexiGenen DNA kit; Qiagen, Germany) was added to each well and mixed thoroughly. After that 150 µL of denaturation buffer and 20 µL of Protease K (10 mg/mL) were added to the reaction mixture. Cells were incubated at 65 °C for 15 min and 600 µL of absolute isopropanol added and thoroughly mixed until the DNA became visible. DNA was collected after centrifugation (Daihan Scientific, Seoul, Korea) at 10,000*g* for 5 min. The supernatant was discarded and the pellet washed with 70 % ethanol. After the liquid was removed by inverting the Eppendrof tube onto a clean piece of paper, 15 µL of hydration buffer was added to dissolve the DNA for 15 min at 65 °C. The resulting 300 ng/µL of DNA (DNA counting by GE healthcare Life Sciences, United Kingdom) was analyzed by electrophoresis on 1.8 % agarose gels containing 0.75 % ethidium bromide. The DNA was mixed with 6× loading dye and the gel was electrophoresed in 0.5xTBE buffer at 90 V for 1 min and 50 V for 1 h. DNA fragmentation was visualized by a UV transilluminator (Vilberlourmat, Germany) and the image was captured.

### Alkylating reaction in vitro

An alkylation reaction of the jujube seed extract was performed to understand the interaction of the extract with DNA resulting DNA damage. The alkylation assay was conducted following the method modified by Machana et al. [30]. NBP (final concentration 20 mM) was prepared in acetonitrile. The buffer solution (pH 4) comprised 0.5 µM of citric acid, 1 µM of boric acid, 4.9 µM of sodium chloride, and 1 M sodium hydroxide adjusted to a volume of 10 mL with deionized water. The mixture of 180 µL NBP and 420 µL buffer solution was prepared and heated to 70 °C for 30 min. The extracts at 2 × IC_50_ concentrations dissolved in acetonitrile were pipetted into the NBP mixture. Then, 120 µL of solution mixture was pipetted and added into a 160 µL ethanol–acetonitrile mixture at a ratio of 1:3. The wavelength of NBP adduct was measured at 580 nm at different time points (5, 15, 30, 45, 60 and 75 min).

### Phytochemicals screening

Phytochemical screening was performed to identify the presence of the main phytoconstituents in 0.025 g of jujube seed extract of each cultivar. Identification of alkaloids, saponins and carbohydrates were tested as per Anandanayaki [35], while identification of flavonoids was conducted as per Bello et al. [36].

#### Alkaloids identification

Jujube seed extracts were dissolved in 500 µL of methanol and centrifuged (Daihan Scientific, Seoul, Korea) at 268.3*g* for 10 min. HCl (1 %, 1 mL) and 1 drop of Dragendorff’s reagent (potassium bismuth iodide) was added to the supernatant. An orange or reddish-brown precipitate indicated a positive test.

#### Flavonoids identification

Methanol (2.5 mL) and 0.5 mL of 10 % NaOH were added to the extracts. A few drop of 1 % HCl was added to 0.5 ml of supernatant. The disappearance of the yellow color indicated the presence of flavonoids.

#### Saponins identification

The extracts were diluted with deionized water to a final volume of 1.5 mL and shaken for 30 min. Permanent foamation indicated the presence of saponins.

#### Reducing sugar identification

Fehling’s solution A was prepared by dissolving 3.5 g of copper II sulfate in deionized water and adjusted to 50 mL. Fehling’s solution B was prepared by dissolving 17.5 g potassium sodium tartrate with 5 g sodium hydroxide in 50 mL of deionized water. The extracts were diluted in 1.5 mL of deionized water, heated with Fehling’s A and B solution (mixture 1:1) for 10 min. An orange-red precipitate indicated a positive result and the presence of reducing sugar.

### Statistical analysis

The results from multiple independent experiments were expressed as mean ± standard deviation. One-way ANOVA was used to test for significant differences between means. *P* < 0.05 was considered statistically significant. The variation between groups was tested by the Tukey range test with IBM SPSS software version 19.0 (SPSS Inc., USA). The concentration- and time-dependence shown in the graphs was visually determined.

## Results and discussion

### Characteristics of seeds

The eight jujube cultivars had diverse seed characteristics (Fig. 1). Generally, the cultivars had different shapes, sizes, and gnarled seed surfaces, with the average width and length being 0.5 cm and 1.6 cm, respectively. The ‘Nomsod’ and ‘Taiwan’ cultivars had the largest seed sizes, while the ‘Kaew’ cultivar had the smallest.

### Antiproliferative effects

The antiproliferative effects of seed extracts from the eight jujube cultivars were evaluated on the leukemic Jurkat cell line, in comparison with the non-cancer Vero cell line, by neutral red assays (Table 1). The cationic neutral red dye can penetrate through cell membranes and lysosomal membranes to attach to anionic charges in the lysosomal matrix of living cells [37].

**Table 1:** Antiproliferative activity of different jujube seed extracts on non-cancer Vero cell line and T cell leukemic Jurkat cell line evaluated by the NR assay for 24 h incubation

Sample	IC_50_ (µg/mL)												Selectivity index
	Vero cell line						Jurkat cell line						
	Water	Ethanol	Dichloro-methane	Ethyl acetate	Chloroform	Hexane	Water	Ethanol	Dichloro- methane	Ethyl acetate	Chloroform	Hexane	
‘Samros’	Inactive	Inactive	Inactive	nd	nd	nd	Inactive	417.7 ± 10.4^b^	Inactive	nd	nd	nd	1.0
‘Bombay’	Inactive	Inactive	Inactive	Inactive	Inactive	Inactive	Inactive	487.9 ± 17.5^a^	Inactive	Inactive	Inactive	Inactive	1.0
‘Apple’	Inactive	Inactive	Inactive	Inactive	Inactive	Inactive	Inactive	Inactive	Inactive	Inactive	Inactive	Inactive	nd
‘Taiwan’	Inactive	Inactive	Inactive	Inactive	nd	inactive	Inactive	232.4 ± 7.8^d^	Inactive	Inactive	nd	Inactive	2.1
‘Nomsod’	Inactive	Inactive	Inactive	Inactive	nd	Inactive	Inactive	333.4 ± 2.8^c^	Inactive	Inactive	nd	Inactive	1.0
‘Jumbo’	Inactive	Inactive	Inactive	Inactive	nd	Inactive	Inactive	312.0 ± 18.3^c^	Inactive	Inactive	nd	Inactive	1.6
‘Kaew’	Inactive	Inactive	Inactive	Inactive	Inactive	Inactive	Inactive	Inactive	Inactive	Inactive	Inactive	Inactive	nd
‘Rianthong’	Inactive	Inactive	Inactive	Inactive	nd	Inactive	Inactive	401.6 ± 9.9^b^	Inactive	Inactive	nd	Inactive	1.2
Melphalan	75.0 ± 2.3							119.1 ± 10.4^e^					0.6

Data presented as IC_50_ values and selectivity index in Jurkat cell line (n = 3)

Means in the same column with different superscript letter(s) are significantly different, P < 0.05 (one-way ANOVA). Inactive means at the extract caused <50 % cytotoxicity even using the maximum concentration at 500 µg/mL

*nd* not determined due to their low yield

The antiproliferative effects were represented as IC_50_ values calculated from linear plots between the concentrations and the %cell viability. Extracts with the maximum concentration (500 µg/mL) that inhibited cell viability by <50 % were classified as inactive extracts [30]. Seeds extracted using water, dichloromethane, ethyl acetate, or hexane inhibited Jurkat cell viability by <50 % even when the maximum concentration of 500 µg/mL was used. Therefore, these solvent extracts were defined as inactive against both Jurkat and Vero cell lines. The ethanolic extract of ‘Taiwan’ (232.4 ± 7.8 µg/mL) exhibited the highest antiproliferative effect, followed by the ethanolic extracts of ‘Jumbo’ (312.2 ± 18.3 µg/mL), ‘Nomsod’ (333.4 ± 2.8 µg/mL), ‘Rianthong’ (401.6 ± 9.9 µg/mL), ‘Samros’ (417.7 ± 10.4 µg/mL), and ‘Bombay’ (487.9 ± 17.5 µg/mL). These six ethanolic extracts showed significant antiproliferative effects compared with the untreated cells (all *P* < 0.001). The antiproliferative effects of individual pairs of ‘Jumbo’ vs*. ‘*Nomsod’ (*P* = 0.322) and ‘Samros’ vs*. ‘*Rianthong’ (*P* = 0.841) did not differ significantly. The chemotherapeutic drug melphalan, used as the positive control, showed nonselective antiproliferative effects on Jurkat cells over Vero cells with IC_50_ values of 119.1 ± 10.4 µg/mL vs. 75.0 ± 2.3 µg/mL, respectively. Although the IC_50_ values of the ethanolic extracts were higher than those of melphalan (i.e., lesser antiproliferative effects) (all *P* < 0.001), the selectivity index of the ethanolic extracts toward Jurkat cells was higher than that of melphalan.

The antiproliferative effects of the ethanolic extracts of the jujube seeds on normal cells were in agreement with previous studies [23, 24, 38]. Ethanolic extracts of *Z. mauritiana* seeds showed antiproliferative effects on HL-60, HeLa, and Molt-4 cell lines without affecting the normal HGF cell line [24], and exerted no cytotoxicity on normal rat liver cells [38]. A chloroform extract of *Z. jujuba* fruit pulp showed antiproliferative effects on MCF-7 and SKBR3 cells without toxicity on normal cells [23]. The present study documented the nonselective cytotoxicity of chloroform seed extracts on Jurkat cells. Owing to the low yields of some chloroform extracts, their cytotoxicity was not determined. Six of the eight ethanolic extracts (i.e., ‘Samros’, ‘Bombay’, ‘Taiwan’, ‘Nomsod’, ‘Jumbo’, and ‘Rianthong’) were selected to determine the mode of cell death.

### Apoptosis morphological changes

**Fig. 2:**
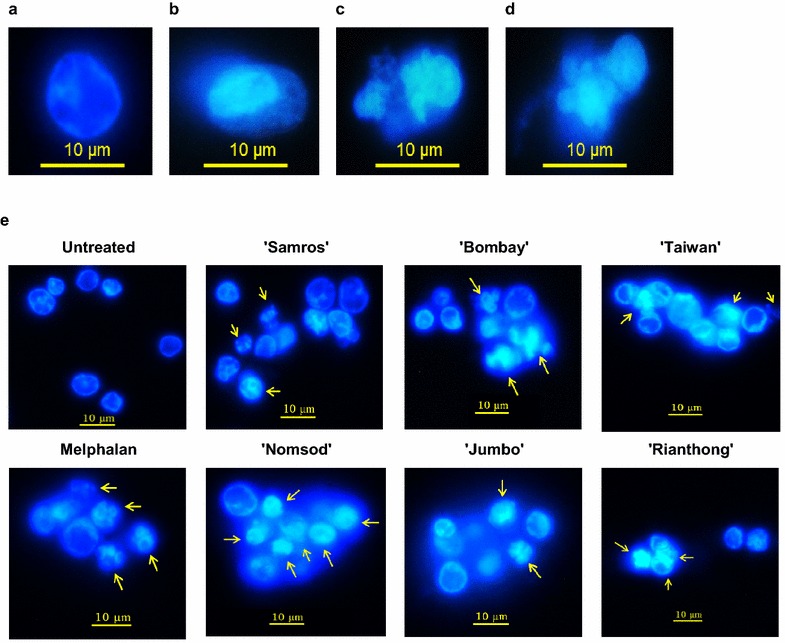
Nuclear morphological changes of apoptotic Jurkat cells stained with DAPI after being treated with 500 µg/mL melphalan (**a**–**d**) and ethanolic jujube seed extracts (**e**) for 24 h and observed under fluorescent microscope at ×100 magnification: (**a**) normal Jurkat nuclei, (**b**) and (**c**) chromatin condensation, and (**d**) apoptotic body formation. Apoptotic nuclei indicated by *arrows*

As shown in Fig. 2a, the normal nuclei of untreated cells had an intact round morphology, whereas the apoptotic nuclei of cells treated with 500 µg/mL melphalan showed chromatin condensation with heterogeneous staining (Fig. 2b, c) and apoptotic body formation (Fig. 2d). The nuclear morphologies of Jurkat cells treated with the ethanolic seed extracts (500 µg/mL) for 24 h were shown in Fig. 2e. The six ethanolic jujube seed extracts induced apoptosis with nuclear morphological changes reminiscent of apoptotic characteristics (i.e., cell shrinkage, chromatin condensation, membrane blebbing, and apoptotic body formation).

### Mode of cell death

The stages of apoptosis can be differentiated from necrotic cell death by flow cytometry using Annexin V-FITC/propidium iodide (PI) staining of cells. Apoptotic lymphocytes are identified by their externalized phosphatidylserine (PS) bound with Annexin V-FITC (green), while necrotic cells are identified by their DNA intercalation of PI (red) [39]. During the early stages of apoptosis, the PS comprising the cell membrane flip to the outside and bind with Annexin V-FITC, showing a cell dot plot in quadrant 4. Damaged or injured cells lose their membrane integrity, including nuclear membrane integrity, such that PI can pass through the nuclear membrane and appear in quadrant 1 (necrotic cells). In the late stages of apoptosis, cells are double-stained and appear in quadrant 2. Normal cells are not stained and appear in quadrant 3.

**Fig. 3:**
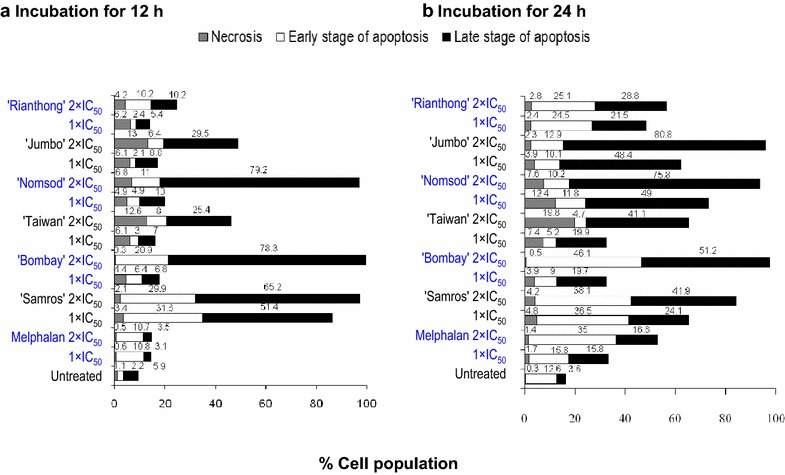
Different modes of cell death determined by flow cytometry. Jurkat cells induced by the ethanolic jujube seed extract and melphalan at 1 × IC_50_ and 2 × IC_50_ at (**a**) 12 and (**b**) 24 h. *Bar graph* represents %cell population at each stage of apoptosis: (*Filled square* ) late, (*open square*) early and (*grey coloured square*) necrosis, respectively. Results are representative of three separate experiments

The ethanolic jujube seed extracts induced cell death in a concentration- and time-dependent manner through diverse modes of cell death (Fig. 3). In general, the extracts induced relatively high late-stage apoptosis and low necrosis compared with melphalan, which induced more early-stage apoptosis. After incubation for 12 h, the 2 × IC_50_ concentrations of ‘Bombay’ (99.2 ± 0.4 %), ‘Samros’ (95.1 ± 1.8 %), and ‘Nomsod’ (90.2 ± 2.7 %) were the first three extracts to induce high total apoptosis (>90 %) in Jurkat cells. The total apoptosis inductions for ‘Bombay’ vs. *‘*Samros’ (*P* = 0.988), ‘Bombay’ vs*. ‘*Nomsod’ (*P* = 0.928), and ‘Samros’ vs*. ‘*Nomsod’ (*P* = 0.050) did not differ significantly. Under the same conditions (12 h, 2 × IC_50_), only ‘Samros’ (29.9 ± 2.6 %) (*P* = 0.004) and ‘Bombay’ (20.9 ± 1.4 %) (*P* < 0.001) significantly induced higher  %early-stage apoptosis than melphalan (10.7 ± 0.4 %). By contrast, the 2 × IC_50_ concentrations of ‘Nomsod’ (79.2 ± 3.0 %) (*P* < 0.001), ‘Bombay’ (78.3 ± 1.6 %) (*P* < 0.001), and ‘Samros’ (65.2 ± 1.2 %) (*P* < 0.001) caused more significant late-stage apoptosis than melphalan (3.5 ± 0.3 %) and the other extracts.

At 24 h, the 2 × IC_50_ concentration of ‘Bombay’ showed the highest  %total apoptosis (97.3 ± 2.9 %) followed by those of ‘Jumbo’ (93.7 ± 2.4 %), ‘Nomsod’ (86.0 ± 1.9 %), ‘Samros’ (80.1 ± 3.2 %), ‘Rianthong’ (53.9 ± 2.6 %), melphalan (51.6 ± 1.1 %), and ‘Taiwan’ (45.8 ± 3.0 %). Only the 2 × IC_50_ concentration of ‘Bombay’ (46.1 ± 2.5 %) (*P* < 0.001) was found to induce early-stage apoptosis significantly more than melphalan (35.0 ± 2.2 %). Furthermore, the first three jujube seed extracts that induced high  %late-stage apoptosis were ‘Jumbo’ (80.8 ± 0.8 %), ‘Nomsod’ (75.8 ± 2.3 %), and ‘Bombay’ (51.2 ± 3.7). The %late-stage apoptosis for ‘Jumbo’ vs. *‘*Nomsod’ did not differ significantly (*P* = 0.599, one-way ANOVA), while the %late-stage apoptosis of ‘Jumbo’ and ‘Nomsod’ *vs.* ‘Bombay’ did differ significantly (*P* < 0.001 and *P* < 0.0.001, one-way ANOVA).

The six ethanolic seed extracts induced apoptosis in a concentration-dependent manner. The ranks of the %total apoptosis-inducing effects at the 2 × IC_50_ concentrations after 24 h of incubation were in the descending order: ‘Bombay’, ‘Jumbo’, ‘Nomsod’, ‘Samros’, ‘Rianthong’, melphalan, and ‘Taiwan’. Apoptosis in the HL-60 cell line was induced by the ethanolic jujube seed extracts in a concentration-dependent manner, and our findings were in agreement with a previous report [24].

The extracts needed to cause less necrotic cell death to achieve effectiveness of their anticancer action. After incubation for 12 h, the 2 × IC_50_ concentrations of ‘Jumbo’ (13.0 ± 1.5 %), ‘Taiwan’ (12.6 ± 4.9 %), and ‘Nomsod’ (6.8 ± 2.4 %) induced relatively high necrotic cell death compared with the other extracts and melphalan (0.5 ± 0.3 %). At 24 h, the 2 × IC_50_ concentrations of the extracts increased necrotic cell death in the descending order: ‘Taiwan’ (19.8 ± 2.0 %), ‘Nomsod’ (7.6 ± 0.8 %), ‘Samros’ (4.2 ± 0.5 %), and ‘Jumbo’ (2.3 ± 0.8 %).

### Caspase activity

Apoptosis can be triggered through both extrinsic and intrinsic pathways, involving caspase cascades that act through initiator caspases and executioner caspases [40, 41]. The initiators are caspase-8 for the extrinsic pathway and caspase-9 for the intrinsic pathway. When the initiator caspases are activated by apoptotic stimuli, the function of the executioner caspase-3 is triggered, which culminates in DNA fragmentation and apoptotic body formation [2, 39, 41]. The present study assessed the activity of both initiator caspases, caspase-8 and caspase-9, to distinguish the extrinsic pathway from the intrinsic apoptotic pathway. Executioner caspase-3 activity was also determined.

**Fig. 4:**
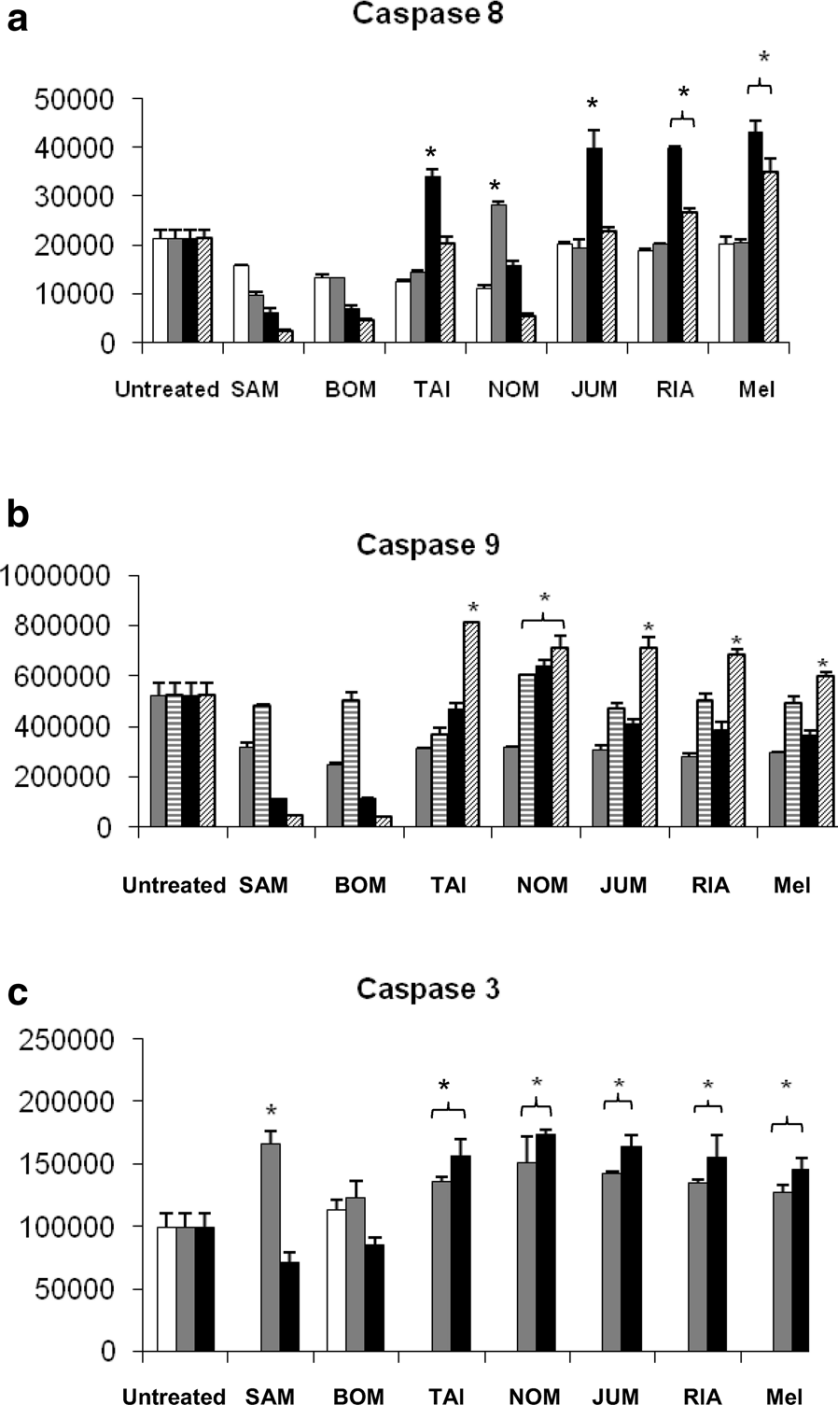
Activity of caspases of the respective ethanolic jujube seed extracts in Jurkat cell line at various times. (**a**), (**b**) and (**c**) represent activity of caspases 3, 8 and 9 after Jurkat cells treated with 2 × IC_50_ of jujube seed extracts at (open square) 15 min, (*grey coloured square*) 30 min, (*lined square*) 60 min, (*filled square*) 180 min, and (*cross lined square*) 360 min (*P < 0.05 compared to untreated cells)

Increased caspase-3 activity was detected at 30 min in Jurkat cells (Fig. 4), suggesting that apoptosis induction by the jujube seed extracts was mediated through the caspase-mediated pathways. ‘Taiwan’, ‘Nomsod’, ‘Jumbo’, ‘Rianthong’, and melphalan all significantly increased caspase-8 and caspase-9 activity compared with untreated cells at different time points (all *P* < 0.001). The increases in caspase-8 activity for ‘Taiwan’ vs. melphalan differed significantly (*P* = 0.006). However, there were no significant differences in the increases in caspase-8 activity for ‘Rianthong’ vs. melphalan (*P* = 0.344), ‘Jumbo’ vs. melphalan (*P* = 0.402), ‘Taiwan’ vs. ‘Rianthong’ (*P* = 0.064), ‘Taiwan’ vs. ‘Jumbo’ (*P* = 0.053), and ‘Rianthong’ vs. ‘Jumbo’ (*P* = 0.999). The increases in caspase-9 activity for ‘Nomsod’ vs. ‘Taiwan’ (*P* = 0.018), ‘Nomsod’ vs. melphalan (*P* = 0.009), ‘Taiwan’ *vs.* ‘Jumbo’ (*P* = 0.019), ‘Taiwan’ vs*. ‘*Rianthong’ (*P* = 0.003), ‘Taiwan’ vs. melphalan (*P* < 0.001), and ‘Jumbo’ vs. melphalan (*P* = 0.009) all differed significantly. In contrast, the increases in caspase-9 activity for ‘Nomsod’ vs. ‘Taiwan’ (*P* = 0.018), ‘Nomsod’ vs*. ‘*Jumbo’ (*P* = 1.000), ‘Nomsod’ vs*. ‘*Rianthong’ (*P* = 0.773), ‘Nomsod’ vs. melphalan (*P* = 0.009), and ‘Jumbo’ vs*. ‘*Rianthong’ (*P* = 0.771) did not differ significantly.

‘Samros’ and ‘Bombay’ did not increase the caspase-8 and -9 activities, although they did activate caspase-3 activity. While activation of caspase-9 was not evident, the ‘Samros’ and ‘Bombay’ extracts were postulated to cleave the caspase-8 substrate earlier than 15 min because of the high activity at 15 min and lower activities at later time points. Activation of caspase-9 and -3 in a concentration-dependent manner in melanoma cells was previously reported for polysaccharides extracted from the fruit part of *Z. jujuba* [42]. The ethanolic extracts of ‘Taiwan’, ‘Jumbo’, ‘Nomsod’, ‘Rianthong’, ‘Samros’, and ‘Bombay’ induced apoptosis via a caspase-mediated pathway in a similar manner to melphalan, but to different extents. The ethanolic extracts of ‘Taiwan’, ‘Nomsod’, ‘Jumbo’, and ‘Rianthong’ and melphalan evidently induced apoptosis via the intrinsic and extrinsic pathways.

### DNA fragmentation

**Fig. 5:**
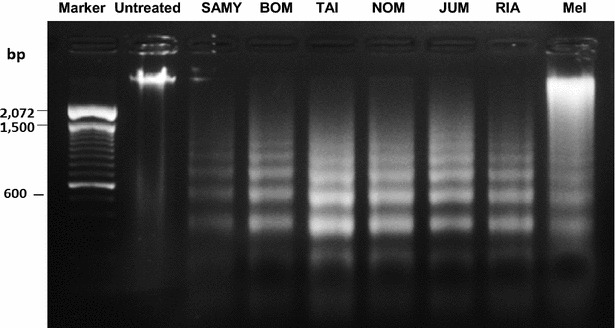
DNA laddering based on gel electrophoresis after the cells were treated with 2 × IC_50_ of ethanolic jujube seed extracts and melphalan for 24 h

DNA fragmentation occurs through the activation of endogenous endonucleases with subsequent cleavage of chromatin DNA into internucleosomal fragments of 180 bp and multiples thereof. Similar to the case for melphalan, all six ethanolic jujube seed extracts at 2 × IC_50_ concentrations clearly exhibited the characteristic DNA ladder formation (Fig. 5).

### Alkylation effects of the selected crude extracts compared with melphalan

An alkylation reaction of the jujube seed extracts was performed to understand the interactions of the extracts with DNA resulting in DNA damage, by testing their alkylating activity. The nitrobenzylpyridine (NBP) assay was used as a model for nucleophilic DNA bases [43–45]. This reaction depends on high temperature and low acidic media to catalyze NBP, which is converted into a blue/violet chromophore product. This specific reaction is positively detected in the presence of electrophilic functional groups or alkylating agents. Only the alkylating drug melphalan led to NBP product formation, suggesting that the DNA damage induced by the jujube seed extracts did not arise from an alkylation reaction. The findings also implied that no electrophilic functional groups were present in the constituents of the jujube seed extracts.

### Phytochemical constituents of the extracts

The phytochemical identification of the ethanolic jujube seed extracts showed that alkaloids predominated in all jujube cultivated species followed by reducing sugars (Table 2). Neither saponins nor flavonoids were found in the cultivars tested. The ‘Apple’, ‘Taiwan’, and ‘Nomsod’ extracts had relatively high amounts of alkaloids, followed by the ‘Samros’, ‘Bombay’, ‘Jumbo’, ‘Kaew’, and ‘Rianthong’ extracts. The ‘Nomsod’ and ‘Rianthong’ extracts had relatively high amounts of reducing sugars.

**Table 2:** Phytochemicals screening of the ethanolic seed extracts of eight jujube cultivars

Phyto-chemicals	Presence of phyto-chemical	Jujube cultivars							
		‘Samros’	‘Bombay’	‘Apple’	‘Taiwan’	‘Nomsod’	‘Jumbo’	‘Kaew’	‘Rianthong’
Alkaloids	Orange or reddish brown precipitate	++	++	+++	+++	+++	++	++	+
Flavonoids	Colorless	−	−	−	−	−	−	−	−
Saponins	Permanent foam	−	−	−	−	−	−	−	−
Reducing sugar	Orange red precipitate	−	−	−	−	+++	−	++	+++

−, absence of phytochemicals; +, presence of phytochemicals; +++, indicates the highest level; +, indicates the lowest level of phytochemical in positive results

Ethanolic extracts of *Z. mauritiana* seeds contained alkaloids, terpenes, flavonoids, saponins, and tannins [46]. The alkaloids exhibited anticancer activity [47]. Alkaloids from *Z. jujuba* seeds (i.e., sanjoine K, zizyphusine, and amphibine) processed a sedative action [48, 49]. The fruit pulp of *Z. jujuba* induced apoptosis in various cancer cells, including breast cancer, liver cancer, and leukemia cells [7, 23]. The bioactive compounds in jujube with the anticancer effects (through induction of apoptotic cell death) were triterpene and betulinic acid [23, 24, 46, 50, 51].

The ethanolic extracts of jujube seeds from different cultivars exhibited diverse inhibition of leukemic Jurkat cell viability. The chloroform extracts and melphalan were toxic toward both Jurkat and Vero cells. The ethanolic extracts selectively caused death in Jurkat cells, but not in normal Vero cells. The ‘Taiwan’ extract exhibited the highest cytotoxicity followed by the ‘Jumbo’, ‘Nomsod’, ‘Rianthong’, ‘Samros’, and ‘Bombay’ extracts. The modes of cell death induced by these six jujube extracts were distinguished and confirmed by different methods. The Jurkat cells treated with these six ethanolic extracts exhibited various stages of apoptotic nuclear morphological alterations similar to those found in Jurkat cells treated with melphalan. The analyses for mode of cell death at 12 h, detected by flow cytometry, revealed that the ‘Bombay’ extract at 2 × IC_50_ induced significantly more apoptosis than the other extracts and melphalan (99.2 ± 0.4 %; *P* < 0.001). At 24 h, the ‘Bombay’, ‘Jumbo’, and ‘Nomsod’ extracts induced apoptotic cell death at 97.3 ± 2.9 %, 93.7 ± 2.4 %, and 86.0 ± 1.9 %, respectively. Although ‘Taiwan’ and ‘Jumbo’ were the first two cultivars possessing high cytotoxicity, they also caused higher degrees of undesirable necrotic cell death. DNA damage was detected in the Jurkat cells treated with the ‘Taiwan’, ‘Jumbo’, ‘Nomsod’, ‘Rianthong’, ‘Samros’, and ‘Bombay’ extracts. This DNA damage did not occur via an alkylation reaction between the extracts and the nucleophilic DNA model, as confirmed by the NBP assays. The Jurkat cellular changes, reflecting apoptosis induced by the seed extracts of the six jujube cultivars, were mediated by caspases. Increased caspase-3 activity was detected after all six extract treatments. The ‘Taiwan’, ‘Nomsod’, ‘Jumbo’, and ‘Rianthong’ extracts induced apoptosis through both extrinsic and intrinsic apoptosis pathways, based on increases in caspase-8 and -9 activities, respectively. The ‘Samros’ and ‘Bombay’ extracts seemed to augment the initiator caspase-8 activity at an earlier time point, but did not induce the intrinsic apoptosis pathway.

## Conclusions

The six active extracts (‘Taiwan’, ‘Jumbo’, ‘Nomsod’, ‘Rianthong’, ‘Samros’ and ‘Bombay’) contained alkaloids and reducing sugars, and induced caspase-dependent apoptosis in human Jurkat leukemia T cells.

## Abbreviations

APP: ‘Apple’ cultivar
BOM: ‘Bombay’ cultivar
JUM: ‘Jumbo’ cultivar
KAE: ‘Kaew’ cultivar
Mel: chemotherapeutic drug melphalan
NOM: ‘Nomsod’ cultivar
RIA: ‘Rianthong’ cultivar
SAM: ‘Samros’ cultivar
TAI: ‘Taiwan’ cultivar

## References

[1] Khazir J, Mir BA, Pilcher L, Riley DL (2014). Role of plants in anticancer drug discovery. Phytochemistry Lett.

[2] Brunelle JK, Zhang B (2010). Apoptosis assays for quantifying the bioactivity of anticancer drug products. Drug Resist Updat.

[3] Yan YH, Gao ZP (2002). Industrialization of Chinese jujube. J Northwest Sci Tech Univ Agri For.

[4] Williams JT, Smith RW, Haq N, Dunsiger Z (2006). From introduction, taxonomy, and history. Fruits for the future 2 (revised edition): ber and other jujubes.

[5] Li JW, Fan LP, Ding SD, Ding XL (2007). Nutritional composition of five cultivars of Chinese jujube. Food Chem.

[6] Saha D, Srivastava SC, Ramani R (2012). Genetic relationships among fruit cultivars and host plants of Indian lac insect in ber (*Ziziphus mauritiana* Lam.) revealed by RAPD and ISSR markers. Indian. J Biotechnol.

[7] Huang X, Kojima-Yuasa A, Norikura T, Kennedy DO, Hasuma T, Matsui-Yuasa I (2007). Mechanism of the anti-cancer activity of *Zizyphus jujuba* in HepG2 cells. Am J Chin Med.

[8] Kumar S, Jawaid T, Dubey SD (2011). Therapeutic plants of Ayurveda; a review on anticancer. Pharm J.

[9] Eley JG, Dovlatabadi H (2002). Permeability enhancement activity from *Ziziphus jujuba*. Pharm Biol.

[10] Peng WH, Hsieh MT, Lee YS, Lin YC, Liao J (2000). Anxiolytic effect of seed of *Ziziphus jujuba* in mouse models of anxiety. J Ethnopharmacol.

[11] Su BN, Cuendet M, Farnsworth NR, Fong HH, Pezzuto JM, Kinghorn AD (2002). Activity-guided fractionation of the seeds of *Ziziphus jujuba* using a cyclooxygenase-2 inhibitory assay. Planta Med.

[12] Heo HJ, Park YJ, Suh YM, Choi SJ, Kim MJ, Cho HY, Shin DH (2003). Effects of oleamide on choline acetyltransferase and cognitive activities. Biosci Biotechnol Biochem.

[13] Lamien-Meda A, Lamien CE, Compaoré MMY, Meda RNT, Kiendrebeogo M, Zeba B, Nacoulma OG (2008). Polyphenol content and antioxidant activity of fourteen wild edible fruits from Burkina Faso. Molecules.

[14] Memarpoor-Yazdi M, Mahaki H, Zare-Zardini H (2013). Antioxidant activity of protein hydrolysates and purified peptides from *Zizyphus jujuba* fruits. J Funct Foods.

[15] Su XS, Chen ZD, Jiao BL, Huang QM, Li WY (2000). Studies on anti-allergic activity of common foodstuffs in China and their constituents. J Southwest Agri Univ.

[16] Suttisri R, Lee IS, Kinghorn AD (1995). Plant-derived triterpenoid sweetness inhibitors. J Ethnopharmacol.

[17] Dahiru D, Obidoa O (2007). Pretreatment of albino rats with aqueous leaf extract of *Ziziphus mauritiana* protects against alcohol-induced liver damage. Trop J Pharm Res.

[18] Siddharth P, Kailash P, Niraj V, Karuna M, Vimal P, Bharadia P, Pundarikakshudu K (2010). Antiulcer activity of methanolic extract of *Ziziphus mauritiana* Stem Bark. Int J Pharm Phytochem Res.

[19] Gupta M, Mazumder UK, Vamsi MLM, Sivakumar T, Kandar CC (2004). Antisteroidogenic activity of the two Indian medicinal plants in mice. J Ethnopharmacol.

[20] Adamu HM, Abayeh OJ, Ibok NU, Kafu SE (2006). Antifungal activity of extracts of some Ca*ssia, Detarium* and *Ziziphus* species against dermatophytes. Nat Prod Radiance.

[21] Dahiru D, Sini JM, John-Africa L (2006). Antidiarrhoeal activity of *Ziziphus mauritiana* root extract in rodents. Afr J Biotechnol.

[22] Mahajan R, Chopda M (2009). Phyto-Pharmacology of *Ziziphus jujuba* Mill-A plant review. Pharmacogn Rev.

[23] Plastina P, Bonofiglio D, Vizza D, Fazio A, Rovito D, Giordano C, Gabriele B (2012). Identification of bioactive constituents of *Ziziphus* jujube fruit extracts exerting antiproliferative and apoptotic effects in human breast cancer cells. J Ethnopharmacol.

[24] Mishra T, Khullar M, Bhatia A (2011). Anticancer potential of aqueous ethanol seed extract of *Ziziphus mauritiana* against cancer cell lines and Ehrlich ascites carcinoma. Evid Based Complement Alternat Med.

[25] Buanong M, Techavuttiporn C, Boonyaritthongchai P, Lichanporn I. Analysis of bioactive compound contents in commercial fruits locally cultivated in Thailand. 2009. http://elibrary.trf.or.th/fullP/RDG5220054//RDG5220054_abstract.pdf.

[26] Taechakulwanijya N, Weerapreeyakul N, Barusrux S, Siriamornpun S (2013). Apoptosis induction effect of three jujube cultivars in HepG2 and Jurkat cell lines. Int J Biosci Biochem Bioinforma (IJBBB).

[27] Siriamornpun S, Weerapreeyakul N, Barusrux S (2015). Bioactive compounds and health implications are better for green jujube fruit than for ripe fruit. J Funct Foods.

[28] Grebenstein C, Choi YH, Rong J, De Jong TJ, Tamis WLM (2011). Metabolic fingerprinting reveals differences between shoots of wild and cultivated carrot (*Daucus carota* L.) and suggests maternal inheritance or wild trait dominance in hybrids. Phytochemistry.

[29] Cherif AO, Messaouda MB, Kaabi B, Pellerin I, Boukhchina S, Kallel H, Pepe C (2011). Characteristics and pathways of bioactive 4-desmethylsterols, triterpene alcohols and 4a-monomethylsterols, from developing Tunisian cultivars and wild peanut (*Arachis hypogaea* L.). Plant Physiol Bioch..

[30] Machana S, Weerapreeyakul N, Barusrux S, Nonpunya A, Sripanidkulchai B, Thitimetharoch T (2011). Cytotoxic and apoptotic effects of six herbal plants against the human hepatocarcinoma (HepG2) cell line. Chinese Med.

[31] Bézivin C, Tomasi S, Lohézic-Le Dévéhat F, Boustie J (2003). Cytotoxic activity of some lichen extracts on murine and human cancer cell lines. Phytomedicine.

[32] De Castro LFP, Zacharias M (2002). DAPI binding to the DNA minor groove: a continuum solvent analysis. J Mol Recognit.

[33] Nasri T, Bosch RR, Voorde S, Fink-Gremmels J (2006). Differential induction of apoptosis by type A and B trichothecenes in Jurkat T-lymphocytes. Toxicol In Vitro.

[34] Hickey TE, Majam G, Guerry P (2005). Intracellular survival of *Campylobacter jejuni* in human monocytic cells and induction of apoptotic death by cytholethaldistending toxin. Infect Immun.

[35] Anandanayaki S. Comparative pharmacognostical studies on selected plants (*Pedalium murex* Roen ex. L. and *Martynia annua* L.). Ph.D. thesis. Tamil University, Department of Environmental and Herbal Science; 2010.

[36] Bello IA, Ndukwe GI, Audu OT, Habila JD (2011). A bioactive flavonoid from *Pavetta crassipes* K. Schum. Org Med Chem Lett.

[37] Shirazi FH, Ahmadi N, Kamalinejad M (2004). Evaluation of northern Iran *Mentha pulegium* L. cytotoxicity. DARU.

[38] Suriyavadhana M, Pakutharivu T (2011). Evaluation of acute and sub-acute toxicity of ethanol extracts of *Entada pursaetha*, *Toddalia aculeata*, and *Ziziphus mauritiana*. World J Life Sci Med Res.

[39] Dai ZJ, Gao J, Ji ZZ, Wang XJ, Ren HT, Liu XX, Wu WY, Kang HF, Guan HT (2009). Matrine induces apoptosis in gastric carcinoma cells via alteration of Fas/FasL and activation of caspase-3. J Ethnopharmacol.

[40] Budihardjo I, Oliver H, Lutter M, Luo X, Wang X (1999). Biochemical pathways of caspase activation during apoptosis. Annu Rev Cell Dev Biol.

[41] McIlwain DR, Berger T, Mak TW (2013). Caspase functions in cell death and disease. Cold Spring Harb Perspect Biol.

[42] Hung CF, Hsu BY, Chang SC, Chen BH (2012). Antiproliferation of melanoma cells by polysaccharide isolated from *Zizyphus jujuba*. Nutrition.

[43] Meier I, Shephard SE, Lutz WK (1990). Nitrosation of aspartic acid, aspartame, and glycine ethylester. Alkylation of 4-(p-nitrobenzyl) pyridine (NBP) in vitro and binding to DNA in the rat. Mutat Res.

[44] Dierickx KME, Journé F, Gerbaux P, Morandini R, Kauffmann JM, Ghanem GE (2009). Improving the spectrophotometric determination of the alkylating activity of anticancer agents: a new insight into the mechanism of the NBP method. Talanta.

[45] Gómez-Bombarelli R, González-Pérez M, Calle E, Casado J (2012). Potential of the NBP method for the study of alkylation mechanisms: NBP as a DNA-model. Chem Res Toxicol.

[46] Mishra T, Bhatia A (2010). Augmentation of expression of immunocytes’ functions by seed extract of *Ziziphus mauritiana* (Lamk.). J Ethnopharmacol.

[47] Saxena M, Saxena J, Nema R, Singh D, Gupta A (2013). Phytochemistry of medicinal plants. J Pharmacogn Phytochem.

[48] Liu J, Chen B, Yao S (2007). Simultaneous analysis and identification of main bioactive constituents in extract of *Zizyphus jujuba* var. *sapinosa* (Zizyphispinosi semen) by high performance liquid chromatography-photodiode array detection-electrospray mass spectrometry. Talanta.

[49] Han BH, Park MH, Park JH (2009). Chemical and pharmacological studies on sedative cyclopeptide alkaloids in some Rhamnaceae plants. Pure Appl Chem.

[50] Pisha E, Chai H, Lee IS, Chagwedera TE, Farnsworth NR, Cordell GA, Brown DM (1995). Discovery of betulinic acid as a selective inhibitor of human melanoma that functions by induction of apoptosis. Nat Med.

[51] Lee SM, Min BS, Lee CG, Kim KS, Kho YH (2003). Cytotoxic triterpenoids from the fruits of *Zizyphus jujuba*. Planta Med.

